# Prevention of Allergy to a Major Cow's Milk Allergen by Breastfeeding in Mice Depends on Maternal Immune Status and Oral Exposure During Lactation

**DOI:** 10.3389/fimmu.2020.01545

**Published:** 2020-07-21

**Authors:** Karine Adel-Patient, Hervé Bernard, François Fenaille, Stéphane Hazebrouck, Christophe Junot, Valérie Verhasselt

**Affiliations:** ^1^Laboratoire d'Immuno-Allergie Alimentaire, Service de Pharmacologie et d'Immunoanalyse, Département Médicaments et Technologies pour la Santé (DMTS), CEA, INRAE, Université Paris-Saclay, Gif-sur-Yvette, France; ^2^Laboratoire du Métabolisme des Médicaments, Service de Pharmacologie et d'Immunoanalyse, Département Médicaments et Technologies pour la Santé, CEA, INRAE, Université Paris-Saclay, Gif-sur-Yvette, France; ^3^Chair of Human Lactology, School of Molecular Sciences, University of Western Australia, Perth, WA, Australia

**Keywords:** breastfeeding, food allergy, prevention, cow's milk, mouse model

## Abstract

**Background:** The high incidence of food allergy in childhood points to the need of elucidating early life factors dictating allergy susceptibility. Here, we aim to address in a mouse model how the exposure to a major cow's milk allergen through breastmilk of mothers with different immune status influences food allergy outcome in offspring.

**Methods:** BALB/cJ future dams were either kept naïve, or sensitized through the oral route using cholera toxin (“orally sensitized”) or through the i.p. route using alum (“i.p. sensitized”), or rendered fully tolerant (oral gavage without any adjuvant) to bovine β-lactoglobulin (BLG). After mating with naïve males and delivery, mothers were orally exposed or not to BLG during the whole lactation. Then, eight groups of lactating mothers were considered: naïve, i.p. sensitized, orally sensitized, or tolerant, each exposed or not during lactation. In order to specifically address breastmilk effects on their allergy susceptibility, pups from naïve-synchronized mothers were cross-fostered by the different groups of treated dams and lactating mothers at delivery. In some experiments, mothers kept their own pups to address a possible *in utero* effect. BLG antigen, BLG-specific antibodies, and BLG-immune complexes were measured in breastmilk from the different lactating mother groups. Allergic sensitization was monitored in 5-weeks old female offspring (*n* = 7–8/group of lactating mothers) by determining BLG-specific antibodies in plasma and splenocytes cytokine secretion after i.p. injections of BLG/alum. Allergic reaction to oral BLG challenge was evaluated by measuring mMCP1 in plasma.

**Results:** Offspring was protected from one allergic i.p. sensitization when nursed by i.p. sensitized mothers, independently of BLG exposure during lactation. Orally sensitized dams conferred protection in offspring solely when exposed to BLG during lactation, while naïve mothers did not provide any protection upon BLG exposure. The levels of protection correlated with the levels of BLG-specific antibodies and BLG-immune complex in breastmilk. There was a trend for decreased sensitization in offspring breastfed by tolerant and exposed mothers, which was not associated with transfer of specific antibodies through breastmilk. Protection provided by nursing by treated/exposed mothers was not persistent after a boost i.p. injection of the progeny and then did not protect them from an allergic reaction induced at this time point. No additional *in utero* effects were evidenced.

**Conclusion:** Our study demonstrates the strong potential of breastmilk to modulate immune response to a major cow's milk allergen in the progeny. It highlights the importance of maternal immune status and of her consumption of the allergen during lactation in dictating the outcomes in offspring. This opens perspectives where modulating maternal immune status might increase the chance of cow's milk allergy prevention in breastfed children.

## Introduction

Immunoglobulin-E (IgE)-mediated food allergies are hypersensitivity reactions against harmless food proteins occurring in predisposed individuals. Instead of a clinically silent immune regulatory response, food allergic people mount inflammatory immune responses driven by Th2 cells upon ingestion of a food allergen (1). This results from an impaired induction of oral immune tolerance toward food antigens or its breakdown. Because the incidence of allergic disease peaks in infancy and childhood, there is a need to identify which early life factors are dictating allergy susceptibility (1).

The perinatal period is a critical period in which both microbiota implantation and type of feeding impact on the maturation of the gut immune system and the epithelial barrier, and thus on the propensity to develop food allergy later in life. Notably, breastmilk might influence immune system development via the transfer of various immunomodulatory molecules directly acting on the epithelial and immune system, or acting via the microbiota, such as regulatory/pro-inflammatory cytokines, miRNA, immunoglobulins, nutrients, but also metabolic products from the microbiota (2–5). Human breastmilk also contains food antigens, which have been ingested by the mother (6–17). While the factors controlling food antigen shedding in breastmilk are poorly identified, the excretion of food antigens, at low doses and over a long period of time after ingestion (>24 h), appears as a natural process. This might have a role in the education of the immune system to environmental antigens to which the newborn will be naturally exposed: actually, as part of the usual diet of the mother, they might correspond to dietary habits of the family.

Mouse studies evidenced that oral administration of ovalbumin (Ova) to naive mice during lactation led to excretion of Ova in milk, which induced partial protection of the progeny from experimental Ova-induced allergic airway inflammation. The protection was antigen-specific and dependent of transforming growth factor-beta (TGF-β) in breastmilk (18). However, the protective effect provided by Ova-exposure during lactation was far more intense and durable if the mothers were first immunized to the allergen. Breastfeeding-induced tolerance then involves the transfer of IgG-Ova complexes to the neonates, their loading through the neonatal receptor for immunoglobulin constant region (FcR) in the gut and the induction of specific Foxp3^+^CD25^+^ regulatory cells (19–21). These observations were further extended to mice models of Ova-induced allergic diarrhea (17).

In order to expand the knowledge on how to prevent food allergy by breastmilk, we aimed to address whether observations obtained with an egg allergen could be extended to the major cow's milk allergen, bovine β-lactoglobulin, a frequent cause of food allergy in infancy. Furthermore, in order to better reflect the human setting, we also aimed to assess the role of the immune status of the mother in this protection. We then considered either naïve, tolerant, moderately sensitized, or highly sensitized mothers who were exposed or not to the food allergen during lactation.

## Materials and Methods

### Mice

Female and male BALB/cJ Rj mice, 4 weeks-old, were purchased from CERJ (Centre d'Elevage René Janvier, Le Genest-Saint-Isle, France), and were housed in filtered cages under normal specific pathogen free husbandry conditions, with autoclaved bedding and sterile water. Mice received a diet deprived of animal proteins in which BLG was not detected using specific immunoassays (22).

### BLG Purification

Native BLG (BLG) was purified from raw cow's milk (non-heated, Ferme de Viltain, Saclay, France) using selective precipitation and chromatography, and further characterized, as previously described (23).

### Sensitization or Tolerization of the Future Dams, and Oral Exposure to BLG During Lactation

**Figure 1:**
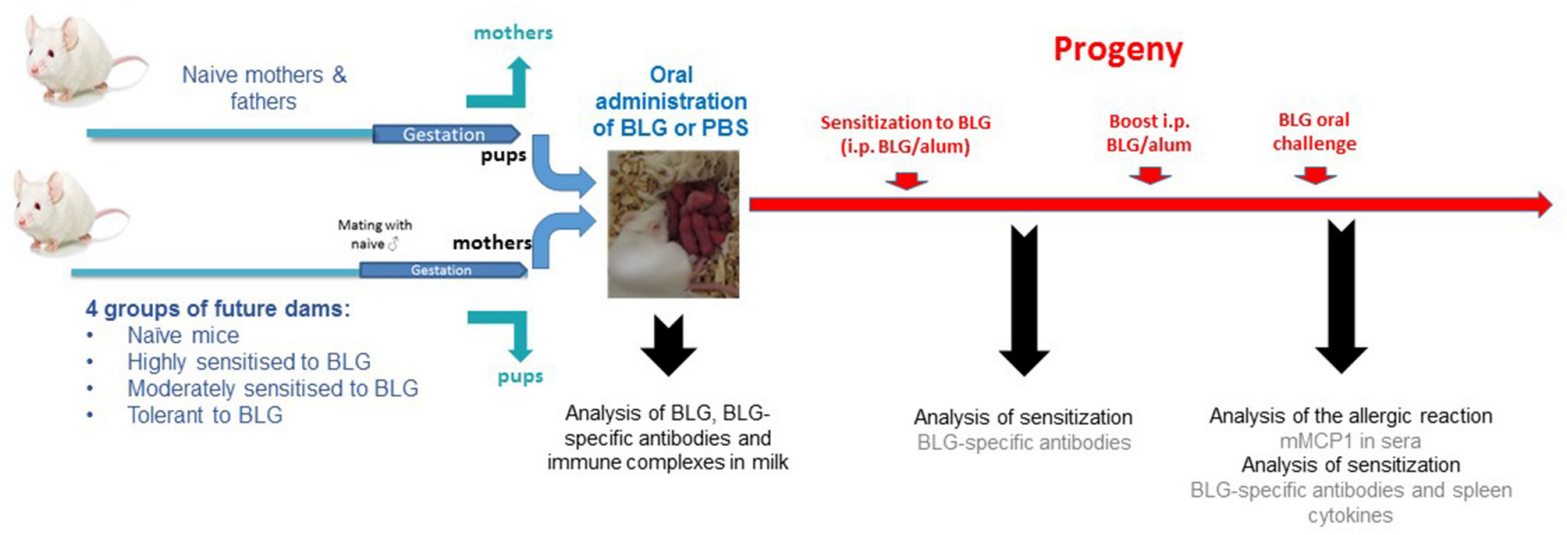
Experimental protocol: BALB/cJ future dams were either kept naïve, moderately (intra-gastric gavage with BLG, and *Cholera toxin*) or highly (intra-peritoneal administration of BLG/alum) sensitized to BLG, or rendered tolerant to BLG (intra-gastric gavage with BLG alone) before mating with naïve males. At delivery, pups of treated mothers were replaced by pups from naïve-synchronized mothers in order to prevent interferences from *in utero* effects. Lactating mothers from each group then received BLG or PBS by i.g. gavage every other day, during the whole period of lactation. Breastmilk was collected 10 days post-partum by pooling stomach contents from 6 to 10 male pups per mother group to assess BLG, BLG specific antibodies, and BLG-immune complexes. The female progeny was then experimentally sensitized by i.p. injection of BLG and Alum when 5 weeks-old and BLG-specific IgE, IgG1, and IgG2a were measured 3 weeks later. A boost injection was performed 21 days after the first i.p. sensitization, and 2 weeks later, all mice were orally challenged with 15 mg of purified BLG. Plasma was collected 60 min later, both to assess allergic sensitization and elicitation of the allergic reaction. Mice were then sacrificed and spleens were pooled to assess *ex vivo* BLG-specific cytokine secretion.

A first group of female mice was highly sensitized when 7 weeks-old by i.p. injection of 5 μg of BLG adsorbed on alum (Alhydrogel 3%, Superfos, Danemark, 1 mg/mouse), with a second injection performed 14 days apart, a model known to induce very high levels of IgE and IgG1 specific antibodies and high Th2 cytokine secretion (24–26) (“i.p. sensitized” mothers, *n* = 15). Another group of female mice was moderately sensitized when 4 weeks-old by performing intra-gastric gavage with 2 mg of BLG mixed with 10 μg of Cholera toxin (Sigma-Aldrich, Saint-Louis, US). Gavaged were repeated once a week for 5 weeks. This model allows inducing specific IgE, IgG1, and Th2 cytokine secretion, but that are far lower than induced by the i.p. route using alum (24) (“orally sensitized” mothers, *n* = 15). A third group of female mice was rendered fully tolerant by repeated gavage with 2 mg of BLG alone when 8 weeks old, a model allowing induction of regulatory T cells that prevent any further sensitization to BLG and any induction of BLG-specific antibodies (26, 27) (“tolerant mothers,” *n* = 15). An fourth group of female mice was kept untreated (naïve mothers, *n* = 15). When 9 weeks old, and 2 days after the last sensitizing/tolerating treatment, all females were mated with age-matched naïve males. Sixty six percent of the females were pregnant, and at delivery, pups from sensitized/tolerated mothers were replaced by pups from naïve-synchronized mothers in order to exclusively assess breastfeeding effect and not the *in utero* effect (Figure 1). Lactating mothers were then exposed or not to 1 mg of BLG by gavage (200 μl/administration, diluted in PBS) every other day starting 48 h after delivery and until weaning. Non-exposed mice received only PBS, so they and their pups had the same handling/stress as in the group of exposed mothers.

In order to assess any additional *in utero* effect, another experiment was conducted in which pups were kept by their respective mother and protocol then performed as before.

### Milk Collection

Breastmilk was collected 10 days post-partum from the stomach of 6–10 male pups per mother group. Males were sacrificed 4 h after gavage of the mother with BLG (or PBS) and stomach content was collected and pooled per mother treatment (2–3 mothers per treatment group). Content was weighted and diluted in two volumes of PBS. After vortexing and centrifugation (10,000× *g*, 10 min, +4°C), supernatants were collected and stored at −20°C until analysis.

### BLG, BLG-Specific Ig Antibodies, and BLG-Ig Immune Complexes in Breastmilk

Enzyme immunometric assays were performed in 96-well microtiter plates (Immunoplate Maxisorb, Nunc, Roskilde, Denmark) using AutoPlate Washer, Microfill dispenser and spectrometer equipments from BioTek instruments, Inc (Avantec, Rungis, France).

BLG antigen and BLG-specific IgG1, IgA, and IgE were quantified in serial dilution of breastmilk samples (from 1/5 to 1/625) as previously described (22, 25, 28). As no standard is available for BLG-specific IgA, results are expressed as absorbance measured at 414 nm.

BLG-IgG1, BLG-IgA, and BLG-IgE immune complexes were assayed on plates coated with IgG purified from rabbit hyperimmunized with BLG. Serial dilutions (from 1/5 to 1/625) of breastmilk samples were performed in immunoassay buffer (0.1 M phosphate buffer, 0.1% bovine serum albumin, 0.01% sodium azide) and applied to coated plates for 18 h at 4°C. After extensive wash (0.01 M phosphate buffer pH 7.4, 0.05% Tween 20), acetylcholinesterase (AChE)-labeled anti-mouse IgE, anti-mouse IgG1, or anti-mouse IgA antibodies were applied for 3 h at room temperature, and solid-phase bound AChE activity was determined by addition of 200 μL/well of Ellman's medium. Absorbance was then measured at 414 nm (25, 28). A positive control of IgG1-BLG immune complex was provided by mixing purified anti-BLG IgG1 monoclonal antibodies (10 ng/ml) with purified BLG (1 ng/ml). No specific IgA-BLG or IgE-BLG immune complexes were detected, whatever the group of lactating mothers considered.

### Allergy to BLG in Offspring (Figure 1)

#### Protocol of Induction of Allergy to BLG

When 5 weeks old, the female offspring nursed by the different groups of mothers (e.g., naïve, naïve exposed during lactation, i.p. sensitized, i.p. sensitized exposed during lactation, orally sensitized, orally sensitized exposed during lactation, tolerant, tolerant exposed during lactation) was sensitized to BLG by i.p. immunization with alum (7–8 mice/group of mothers). Plasma samples were collected 20 days later to assess allergic sensitization. To assess the persistency of any effects, a boost injection was performed 21 days after the first i.p. sensitization, and 2 weeks later, all mice were orally challenged with 15 mg of purified BLG. Plasma was collected 60 min later, both to assess allergic sensitization and elicitation of the allergic reaction.

#### Evaluation of Allergic Sensitization

BLG specific IgG1, IgE, and IgG2a were quantified on plasma samples collected from progeny using BLG-coated microtiter plates (25, 26, 29). Due to high IgG concentrations that might mask epitopes for IgE binding after the i.p. boost (25), a reverse assay using anti-mouse IgE coated plates and AChE-labeled BLG was also performed. Results are then expressed as mAU_414nm_ (no standard available). Non-specific binding was assessed using plasma samples collected from naïve progeny (non-sensitized progeny from naïve and non-exposed mothers).

#### Evaluation of Allergic Reaction

Mouse Mast Cell Protease-1 (mMCP1), a specific marker of intestinal mast cell activation (Moredun Scientific Limited, Midlothian, UK), was assessed on plasma samples collected 60 min after BLG oral challenge following provider recommendations.

#### Splenocytes Cytokine Secretion

After oral challenge, spleens were harvested and pooled within each mother treatment group. After lysis of red blood cells (180 mM NH_4_Cl, 17 mM Na_2_EDTA) and several washes, splenocytes were resuspended in RPMI-10 (RPMI supplemented with 10% fetal calf serum, 2 mM L-glutamine, 100 U penicillin, 100 μg/mL streptomycin**—**all from Gibco). Cells were incubated in 96-well culture plates (10^6^ cells/well) in the presence of BLG (20 μg/mL), RPMI-10 (negative control), or concanavalin A (1 μg/mL, positive control) for 60 h at 37°C and 5% CO_2_. Each culture conditions were performed in duplicates. Culture were centrifuged (300× *g*, 10 min) and supernatants were collected and stored at −80°C until further assay for cytokines using multiplexed kits and apparatus from Biorad (Bio-Plex Pro™ Mouse Group I and Bioplex100™ apparatus; Marnes la Coquette, France).

### Statistical Analysis

Due to the number of animal included per group (*n* < 30) and as data were not normally distributed, we used non-parametric tests. Presence of differences between groups was first tested using non-parametric Kruskall-Wallis test, and *p*-values calculated using Monte Carlo simulation (10,000 permutations). Pairwise multiple comparison was then performed using Conover-Iman testing, including Bonferroni correction for multiple testing. When specified, Mann Whitney test was additionally performed between two specific groups. All statistical analysis were performed using XLSTAT™ 2019 (version 2.3, Addinsoft, France).

## Results

### The Transfer of BLG Antigen, BLG-Specific Antibodies, and BLG-Immune Complexes Into Breastmilk Depends on Maternal Immune Status

**Figure 2:**
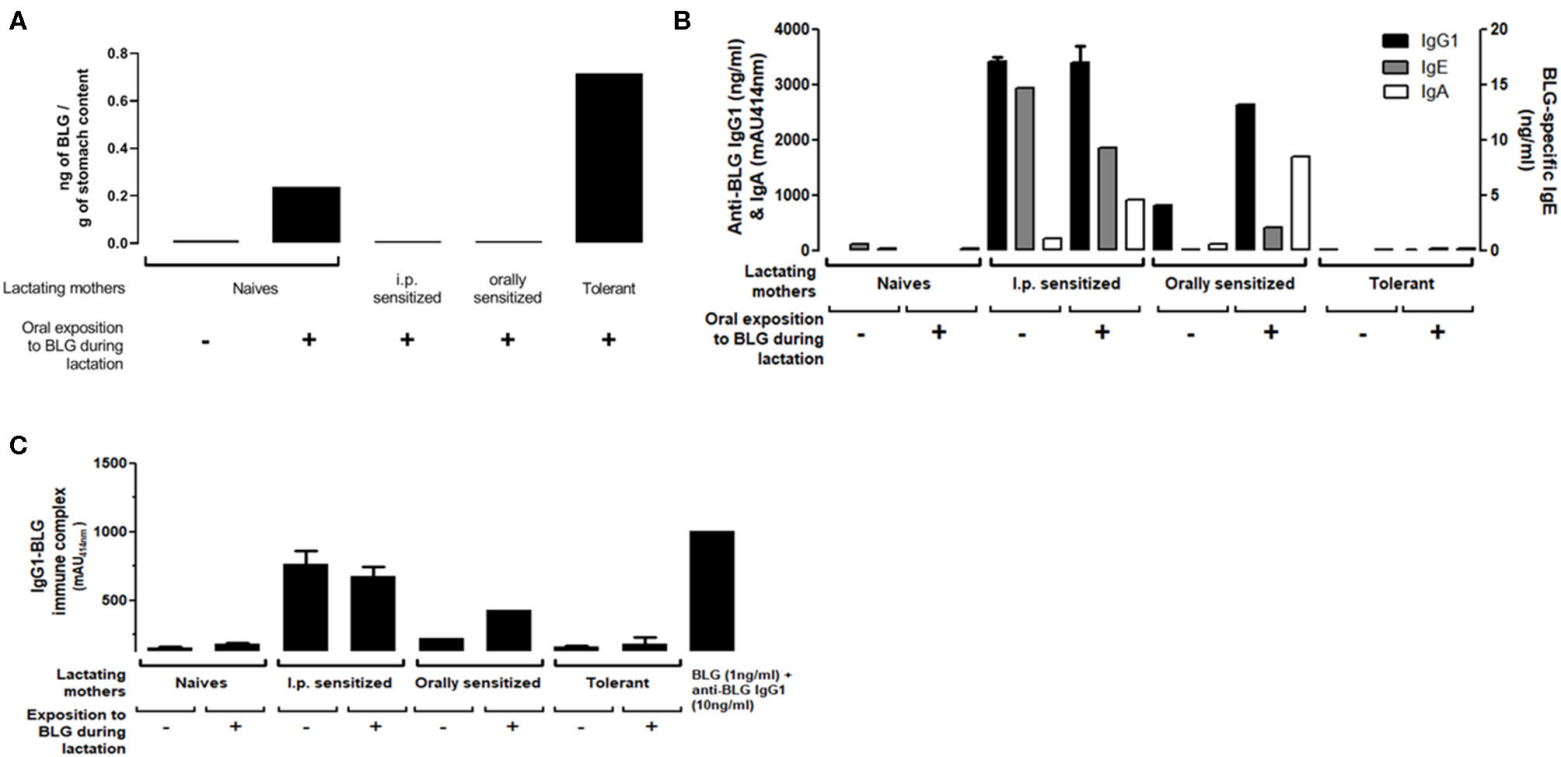
**(A)** BLG antigen, **(B)** BLG-Specific IgG1 (black bars), IgA (white bars) and IgE (gray bars) and **(C)** IgG1-BLG immune complexes detected in milk collected 10 days post-partum from naïve, sensitized or tolerant mothers exposed (+) or not (–) to 1 mg of BLG every other day during lactation. Assays were performed on stomach contents collected from pups 4 h after the exposition of their fostered mothers. For BLG-specific IgA and IgG1-BLG immune complexes, absorbance signals (mAU_414nm_) obtained at the 1/25 dilution are reported. A positive control of IgG1-BLG immune complex was provided by mixing purified anti-BLG IgG1 monoclonal antibodies with purified BLG (right bar).

Using a BLG-specific sandwich immunoassay, we detected BLG in milk collected from naïve or tolerant mothers who had been exposed to BLG during lactation (Figure 2A). In contrast, we could not detect BLG in milk collected from orally (moderate sensitization) or i.p. (high sensitization) sensitized mothers orally exposed to BLG during lactation.

BLG-specific IgE, IgG1, and IgA (Figure 2B) and BLG-IgG1 immune complexes (Figure 2C) were undetectable in milk from naïve or tolerant mothers, whether they had been exposed or not to BLG during lactation. In milk from orally sensitized mothers, BLG-specific Ig and immune complexes were detected and their levels increased with BLG exposure during lactation. I.p. sensitized mothers had the highest levels of BLG-specific Ig and BLG immune complexes. They were not further increased by BLG-exposure during lactation, except for the BLG-specific IgA.

### Protection From i.p. Sensitization to BLG in Offspring Depends on Maternal Immune Status and Oral Exposure to BLG During Lactation

**Figure 3:**
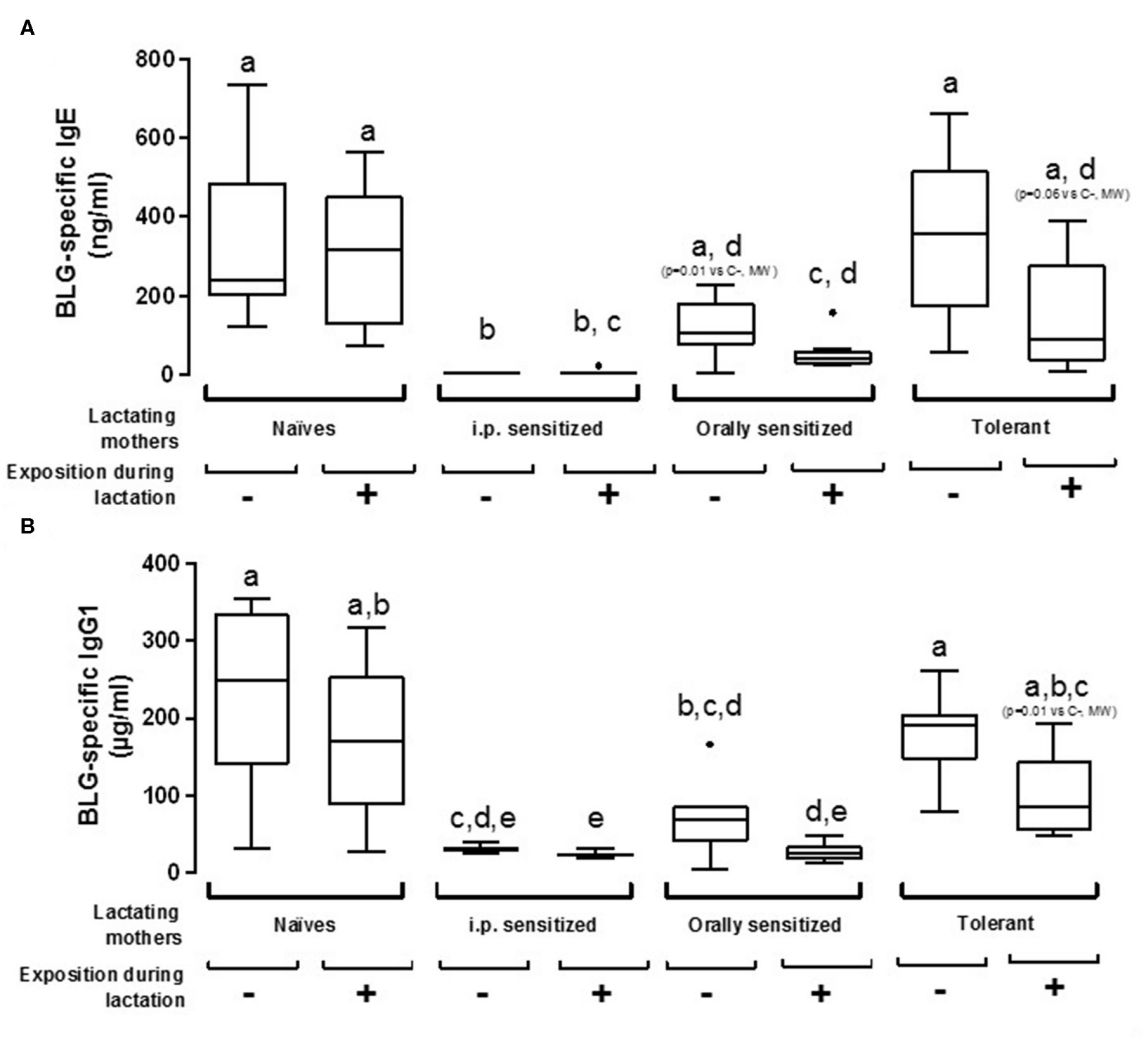
Plasma BLG-Specific IgE **(A)** and IgG1 **(B)** antibodies induced after one i.p. sensitization with BLG/alum in pups fostered by naïve or pre-natally sensitized or tolerated mothers, further orally exposed (+) or not (–) to BLG during lactation. Tukey box and whiskers from 7 to 8 mice/groups are shown. Statistical analysis evidenced differences between groups (*p* < 0.0001, non-parametric Kruskall Wallis test). Pairwise multiple comparisons were then performed using Conover-Iman testing, including Bonferroni correction for multiple testing. Groups indicated with different letters are different from each other's (*p* < 0.05). *p*-values obtained using additional testing against control (naive and non-exposed mothers) by Mann Whitney test are also indicated between brackets.

The susceptibility of offspring from various mothers' group to be sensitized to BLG was first assessed by measuring BLG-specific antibodies after one i.p. sensitization with BLG in Alum. Mice fostered by naïve mothers exposed or not to BLG during lactation demonstrated comparable sensitization levels, as evidenced by comparable concentrations of BLG-specific IgE and IgG1 (Figure 3). A trend in decreased BLG-specific IgE and IgG1 antibodies concentrations were evidenced in progeny fed by tolerant and exposed mothers (*p* = 0.06 and *p* = 0.01, respectively, using Mann-Whitney test and when compared to naïve non-exposed mice), whereas no effect was evidenced in absence of exposure. Progeny fostered by orally sensitized mothers were significantly protected from sensitization only if mothers were exposed to BLG during lactation, although a trend in decreased BLG-specific IgE concentrations was also noticed without this exposure (*p* = 0,01 vs. naive non-exposed mother, using Mann-Whitney test). In contrast, progeny fostered by i.p. sensitized mothers were fully protected from sensitization, whether exposed or not during lactation, as evidenced by the nearly absence of specific IgE and the very low concentrations of BLG-specific IgG1.

### Protection Is Not Persistent After a Boost i.p. Injection of the Progeny With BLG and Then Does Not Protect the Progeny From Allergic Reaction Elicitation

**Figure 4:**
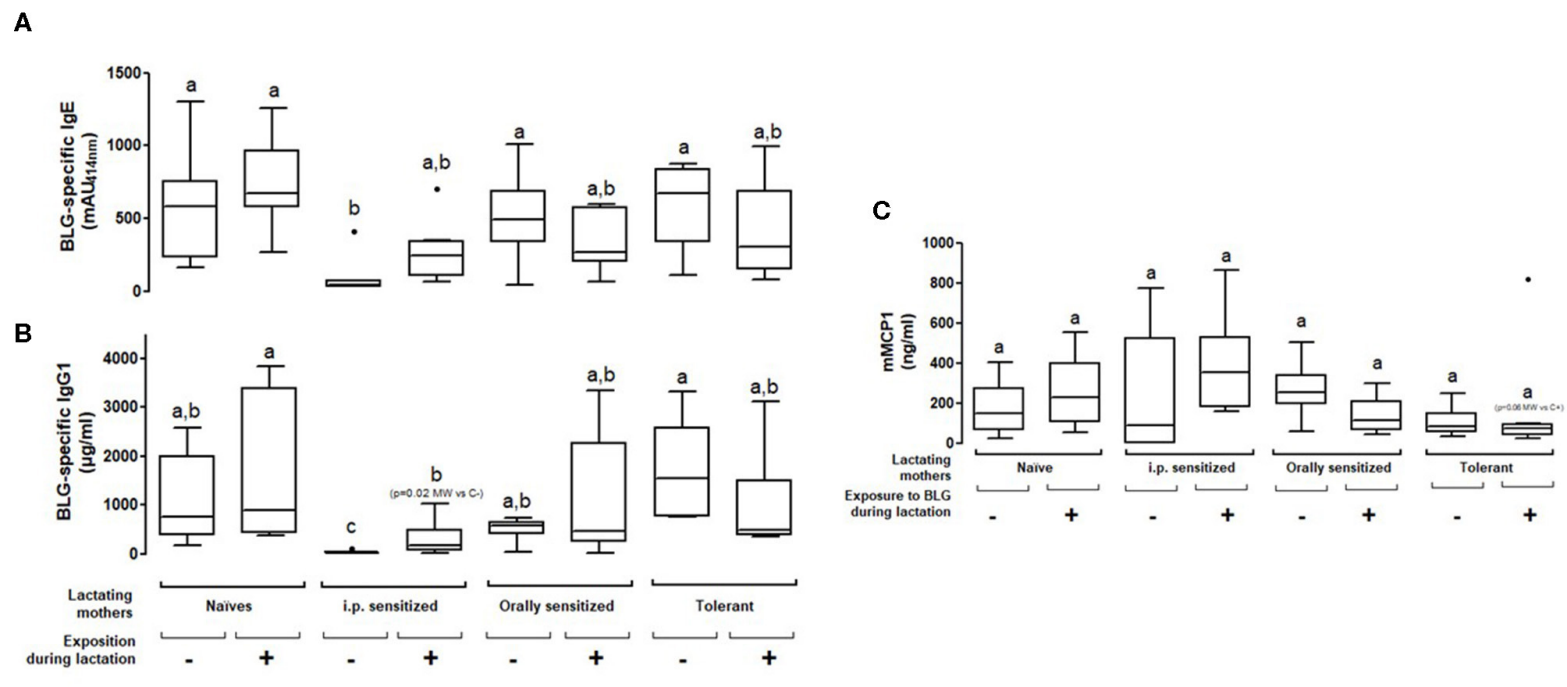
Plasma BLG-Specific IgE **(A)**, IgG1 **(B)**, and mMCP1 **(C)** induced after two i.p. sensitizations and then an oral food challenge with BLG in pups fostered by naïve or prenatally sensitized or tolerized mothers, further orally exposed (+) or not (–) to BLG during lactation. Tukey box and whiskers from 7 to 8 mice/groups are shown. Statistical analysis evidenced differences between groups (*p* = 0.002 for IgE and *p* = 0.038 for mMCP1, non-parametric Kruskall Wallis test). Pairwise multiple comparisons were then conducted using Conover-Iman testing, including Bonferroni correction for multiple testing. Groups indicated with different letters are different from each other's (*p* < 0.05). *p*-values obtained using additional testing against controls by Mann Whitney test are also indicated (MW).

We further assessed the persistency of the prevention from sensitization observed in the progeny nursed by the different treated/exposed groups of mothers by performing an additional i.p. immunization with BLG and alum and an oral BLG challenge. A significant decrease of BLG-specific IgE concentrations was only observed in the progeny fostered by the i.p. sensitized exposed or non-exposed mothers as compared to naïve non-exposed mothers (Figures 4A,B). However, this protection from systemic sensitization was not associated with a reduced allergic reaction as shown by comparable levels of mMCP1 in all the groups (Figure 4C). The only group that tended to be protected from the elicitation of an allergic reaction was the progeny fostered by tolerant and exposed mothers (*p* = 0.06 using the Mann-Whitney test). No difference of IgG2a concentrations was observed between the different groups of sensitized progeny (data not shown).

### Cellular Response in the Progeny Evidenced Modulated Cytokines Profiles

We then assessed cellular immune responses in the sensitized progeny mice by analyzing cytokine secretion at the end of the experimental protocol. No cytokine secretion was observed after culture with media alone, and Concanavalin A-stimulation led to comparable cytokine secretions in the different groups of mice (not shown). When splenocytes were cultured with BLG, increased secretion of Th1 (IFNγ) and Th17 (IL-17) cytokines was noticed in cells from progeny fostered by naïve mothers exposed during lactation to BLG compared to all the other groups (Supplementary Figure 1). A trend in increased BLG-induced Th2 cytokines IL-5 and IL-13 secretion was noticed in splenocytes from progeny fed by non-exposed and sensitized mothers, either i.p. or orally, which was however not associated with higher humoral responses (Figures 3, 4). Conversely, exposure during lactation greatly decreased BLG-induced Th2 cytokine secretion in those groups. Feeding by tolerant mothers also rather led to a decreased Th2 cytokine secretion, whatever the exposure during lactation. Comparable results were obtained for Th2 cytokines and IL-10.

### Absence of Additional *in utero* Effects

**Figure 5:**
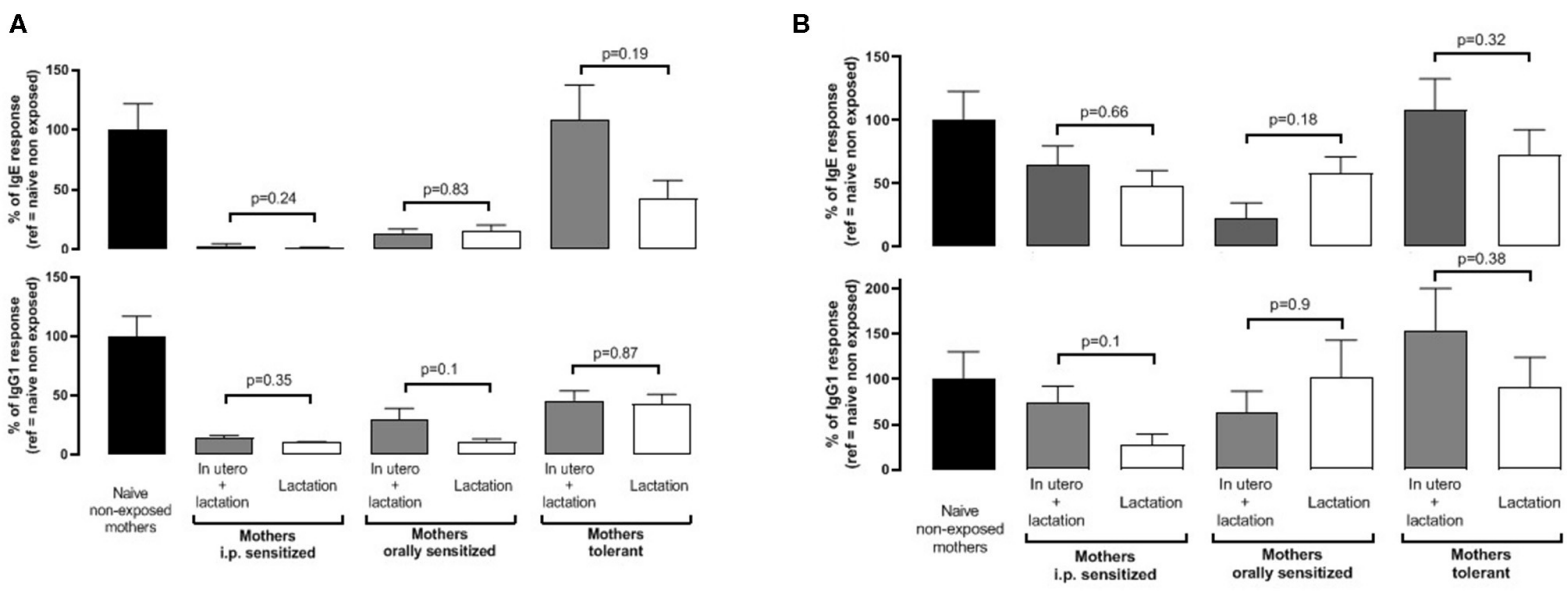
Pups were born and fed by prenatally sensitized (i.p. or orally) or tolerant mothers, further orally exposed during lactation (*in utero* + lactation), or pups from naïve mice were fostered by prenatally sensitized or tolerant mothers further orally exposed during lactation (*n* = 5–12/group). BLG-specific IgE (top panel) and IgG1 (bottom panel) were assayed in plasma from progeny after one **(A)** and two **(B)** i.p. sensitizations with BLG/alum. Specific antibodies are expressed as percentage, with the mean value obtained in naive mothers not exposed during lactation used as a reference (100%). Immune response was compared between “*in utero* + lactation” and “lactation” groups for a same mother pre-treatment, and *p*-values are indicated (Mann Whitney test).

We finally aimed to assess if *in utero* events might provide additional protective effects in the progeny. Therefore, we compared sensitization in pups nursed by their own mothers vs. pups from naïve-synchronized mothers that were cross-fostered by mothers from the different groups. All mothers were exposed to BLG during the whole breastfeeding period. We found similar IgE and IgG1 responses after the first sensitization (Figure 5A) and after the boost injection (Figure 5B) in pups form the mothers fostering their own progeny (“*in utero* + lactation”) or progeny from naïve-synchronized mothers (“lactation”).

## Discussion

New epidemiological and interventional studies demonstrate the food allergy preventive effect of early introduction of some allergens in infant diet such as egg and peanut (LEAP and EAT studies, G. Lack and G. du Toit) (30–32). In contrast, the interventional introduction of cow's milk in the diet of breast-fed infant after 3 months is not associated with protection (33). This is in line with the non-interventional large study from Katz and coworkers that evidenced the highest cow's milk prevalence in infants for who regular exposure to cow's milk protein was withheld until the age of 4–6 months (34). In parallel, studies evidenced that regular introduction of cow's milk formula in the first 2 weeks (34) or first 3 months (35) while pursuing breastfeeding might allow protection. In these studies, no or few information is available on the mother immune status and cow's milk consumption while breastfeeding. Yet, oral exposure in the mother during lactation might already have a significant impact on the breastfed progeny; it has been evidenced recently that early peanut introduction (<12 months) is associated with protection only if the mother consumed peanut while breastfeeding (32). This highlights the need to better understand the way to maximize the chance of food allergy prevention. Here, we then aimed to determine how both the immune status of the mother and her ingestion of a clinically-relevant cow's milk allergen during breastfeeding will impact the allergic outcome in the progeny. Using a mother-child mouse model, we found that these factors do have a major impact on sensitization susceptibility in offspring. Effect on sensitization ranged between nihil for naïve mothers exposed to BLG to a very potent protection for i.p. sensitized mothers (highly sensitized) ingesting or not BLG during lactation. Tolerant mothers and orally immunized (moderate sensitization) mothers induced some protection from sensitization but only when exposed to BLG during lactation. No additional *in utero* effect was evidenced in our experimental set up.

Actually, when we administered BLG to naïve BALB/cJ lactating mothers, we detected BLG in milk collected on D10 but we could not evidence any significant effect on sensitization of the progeny. Our results are not in line with all those obtained following the same experimental schedule and using Ova as a model allergen (18, 20, 36), although others did not evidence protection and even demonstrated enhanced sensitization in the progeny in similar models (37). Importantly, a mother ingesting a food is most of the time not naïve to this food: she is either tolerant, or sensitized, or allergic and the mothers then produce antibodies (IgGs, IgE, IgA) and have T cells (Treg, T helper) specific to the food antigens. Although a high inter-individual variability was noticed, Ova specific IgG and IgA were detected in more than 95% of transition breastmilk from the French birth cohort EDEN, whereas Ova was detected in only 50% of the samples (17). In the present study, we evidenced that sensitization level of the mothers (naïve, moderately (oral) or highly (i.p.) sensitized) determines the concentrations of IgG, IgE, and IgA specific antibodies in milk, and these increase upon oral exposure in the moderately exposed mothers. The concentrations of antibodies were associated with level of protection in the progeny. This is in line with different studies in human that suggest a protective role of high concentrations of breastmilk specific antibodies on child sensitization, and that exposure of the mother to the food allergens during lactation might increase their concentrations (38, 39).

However, immune complexes might be even more efficient than specific antibodies to protect the progeny, as evidenced in the Ova-model in which breastfeeding-induced tolerance by immunized mothers relies on the transfer of IgG-Ova complexes to the neonates (19, 21). Although immune complexes were not assessed in most of the previous cited studies in humans, IgG and IgA immune complexes with gliadin (9) and peanut allergens (13) were evidenced in human breastmilk. Moreover, oral administration of human breastmilk containing peanut allergens (free and complexed) before weaning induced partial protection from sensitization in a mouse model (13). In the present study, BLG-immune complexes levels are related with level of protection in the progeny. Exposure to the allergen is required to detect immune complex in breastmilk in the orally sensitized mothers, whereas exposure to BLG in the BLG/Alum model is dispensable. This might result from the deposit effect of alum allowing progressive release of the antigen, then available for forming immune complexes independently of oral exposure. Although we did not absolutely prove the direct causal role of Ig and immune complex on protection from sensitization, which might be a limitation of our study, all these results suggest that, in the human condition, oral exposure to the allergen during breastfeeding might be critical to form the Ig-immune complexes necessary to induced efficient protection in the progeny.

Another interesting point is that BLG was not detectable in the BM from sensitized and exposed mothers, whereas we were able to detect BLG in the BM from naïve or tolerant mothers exposed during lactation. As BLG detection relied on the use of an immunometric assay, BLG might not be detectable in the former milks due to a masking effect of specific antibodies present in the breastmilk and/or the presence of BLG mainly as immune complexes. The fact that not all mothers were found to be excretors in various studies might result from the same masking effect (7–12, 14–17). This might then imply that all mothers are actually excretors of allergens in their BM, but as a free and/or complexed form depending on the levels of exposure. This should be taken into account in the association studies relating allergen concentrations in breastmilk and allergic outcome in the progeny.

In our mouse model, tolerant mothers tended to protect offspring from sensitization when exposed to BLG during lactation and this protection was not associated with Ig levels or presence of immune complexes in breastmilk. This suggests that other mechanisms, such as transfer in breastmilk of specific immune cell or immuno-suppressive cytokines, might also be involved in the transfer of protection. The actors and mechanisms involved in the protection provided by tolerant and exposed mothers clearly need additional studies.

It is worth noting that sensitization levels and mMCP1 concentrations after the OFCs were not directly correlated. This may be explained by difference in mast cell density and FceRI expression and will require further investigation.

Finally, another point is that the induction of protection we observed with BLG appears to be less efficient than that observed for Ova, despite the same experimental schedule applied (18–21). Notably, we could not evidence any protective effect of exposure to BLG via breastmilk from naïve mothers, and the protection provided by i.p. sensitized mother did not protect progeny from allergic reaction. These observations suggest that, in addition to the immune status of the mother, the nature of the food allergen itself might be important in dictating the possibility to induce oral tolerance in early life. This is also reflected in epidemiological and interventional studies demonstrating the prevention of food allergy by early introduction of some allergens in the diet such as egg and peanut while this was not observed for other allergens such as cow's milk (33). Future work is needed to elucidate which additional approaches are necessary for successful persistent induction of immune tolerance and prevention of allergic disease to cow's milk allergens in early life. Offspring exposure to BLG after weaning might be required, as suggested for peanut (32). Other strategy might also include supplementation in TGF-β in formula given after weaning (17).

In conclusion, our study demonstrated the strong potential of breastmilk to modulate in the long term the immune response to food allergens in offspring. This protective effect is associated with the excretion of the food allergens and immune factors in breastmilk, some of which are increased by exposure to the allergen during lactation. Future studies will need to address whether early immune modulation to cow's milk allergen by exposure through breastmilk might lead to a more successful cow's milk allergy prevention by early introduction in child.

## Data Availability Statement

The raw data supporting the conclusions of this article are available from the corresponding author to any qualified researcher on reasonable request.

## Ethics Statement

All animal experiments were performed according to European Community rules of animal care and with authorization N° 91–368 of the French Veterinary Services. All experiments were covered by agreement no. 2009-DDSV-074 from the Veterinary Inspection Department of Essonne (France).

## Author Contributions

KA-P and VV designed the whole study, analyzed, interpreted the data, and wrote the manuscript. KA-P performed the experiments. HB and SH help to perform some experiments and critically revised the manuscript. FF and CJ critically revised the manuscript. All authors approved the submitted version.

### Conflict of Interest

The authors declare that the research was conducted in the absence of any commercial or financial relationships that could be construed as a potential conflict of interest.
