# Supplementary material for: Prevention of Allergy to a Major Cow's Milk Allergen by Breastfeeding in Mice Depends on Maternal Immune Status and Oral Exposure During Lactation
Source: Front Immunol. 2020 Jul 21;11:1545. doi: 10.3389/fimmu.2020.01545 (PMC7396486; doi:10.3389/fimmu.2020.01545)

Prevention of allergy to a major cow’s milk allergen by breastfeeding in mice depends on maternal immune status and oral exposure during lactation

**Supplementary figures**

**Supplementary figure 1:** BLG-specific cytokine secretion by spleen cells from fostered progeny experimentally sensitized to BLG. To increase readability, IL-5 and IL-13 are plotted on the right axis: be careful that corresponding concentrations are far higher than that observed for IFNγ, IL-17 and IL-10 represented on the left axis. No statistical analysis was performed as results represent single determinations performed on duplicates cell cultures from pooled organs.


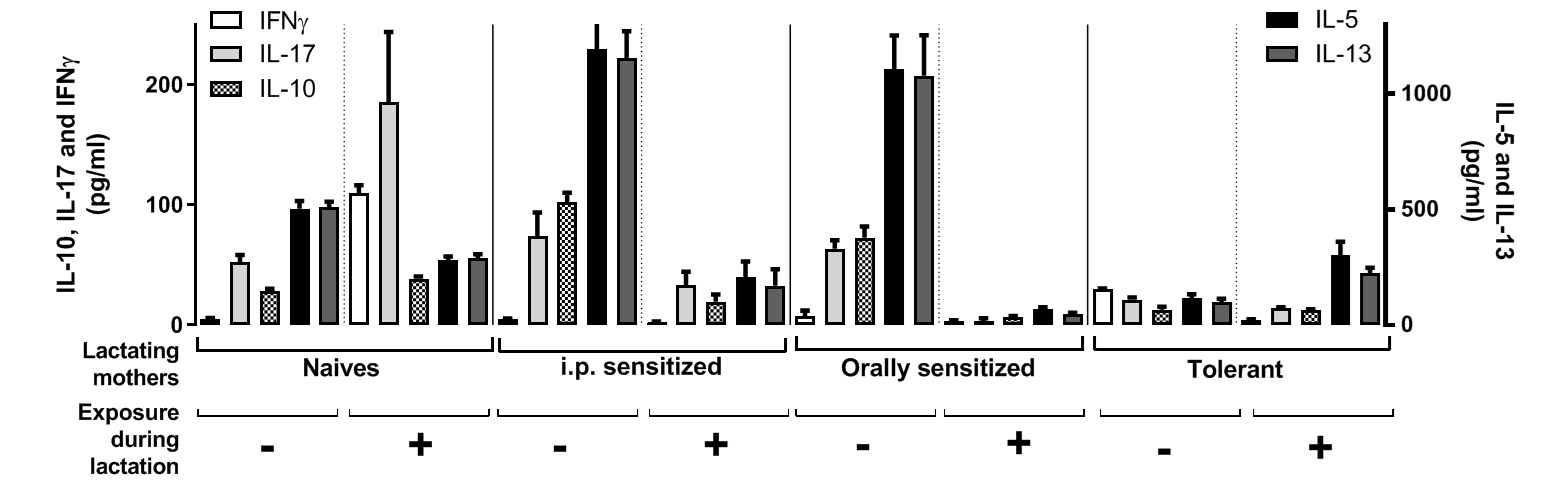

Supplement: Supplementary file 1 [file Data_Sheet_1.docx]
